# Supplementary material for: Dipyridylmethane Ethers as Ligands for Luminescent Ir Complexes
Source: Molecules. 2021 Nov 26;26(23):7161. doi: 10.3390/molecules26237161 (PMC8659258; doi:10.3390/molecules26237161)
Supplement: Supplementary file 1 [file molecules-26-07161-s001.zip › molecules-1448681-supplementary.pdf]

molecules

SUPPLEMENTARY MATERIALS

Dipyridylmethane ethers as ligands for luminescent Ir complexes

Giorgio Volpi[1*], Claudio Garino[1], Roberto Gobetto[1] and Carlo Nervi[1]

[1] Department of Chemistry, University of Turin, via Pietro Giuria 7, 10125 Torino, Italy;
* Correspondence: giorgio.volpi@unito.it

General procedure to ether inermediates ***1***-***3***

A suspension of sodium hydride in dry THF (8 mL) was added to a solution of di(pyridin-2-yl)methanol (**L0**) in dry THF (5 mL) under nitrogen at ambient temperature. After 1 h, when the evolution of hydrogen gas ceased, the resulting dark blue solution was transferred to a solution of corresponding halogenated reagent (Scheme S1) in dry THF (5 mL). The reaction mixture was stirred at ambient temperature for 12 h, and the reaction was terminated by the addition of saturated aqueous NH_4Cl (8 mL). Extraction with CH_2Cl_2 (4×10 mL) followed by washing of the organic phase with saturated aqueous NH_4Cl (5 mL), drying (Na_2SO_4), and evaporation of the solvent gave a crude product which was chromatographed (eluent CH_2Cl_2-CH_3OH 98-2). Reaction yields are reported in Table S1.

Table S1. Synthesis of ether intermediates **1–3**.

Intermediate	R-X		**L0**	NaH	Product	Yield
**1**	3-bromoprop-1-ene	0.350 mL (4.04 mmol)	709 mg (3.38 mmol)	462 mg (19.25 mmol)	277.3 mg (1.23 mmol)	32%
**2**	3-bromoprop-1-yne	1190 mg (10 mmol)	620 mg (3.33 mmol)	400 mg (16.6 mmol)	537 mg (2.40 mmol)	72%
**3**	1-(bromomethyl)-4-iodobenzene	955 mg (3.22 mmol)	500 mg (2.68 mmol)	312 mg (13 mmol)	902mg (2.24mmol)	83%

1: *2,2′-(allyloxymethylene)dipyridine*

^{1}H NMR (400 MHz, $CDCl_3$) δ (ppm): 8.51 (d, J = 4.98 Hz, 2H), 7.82 (d, J = 8.20 Hz, 2H), 7.64 (t, J = 7.76 Hz, 2H), 7.14 (t, J = 6.15 Hz, 2H), 5.71-5.61 (m, 1H), 5.04 (d, J = 14.2 Hz, 1H), 4.95 (d, J = 13.6 Hz, 1H), 3.17 (d, J = 7.03 Hz, 2H).

2: *2,2′-((prop-2-ynyloxy)methylene)dipyridine*

^{1}H NMR (400 MHz, $CDCl_3$ δ (ppm): 8.56 (d, J = 5.12 Hz, 2H), 7.69 (t, J = 7.76 Hz, 2H), 7.59 (d, J = 7.91 Hz, 2H), 7.18 (t, J = 6.15 Hz, 2H), 5.89 (s, 1H), 4.27 (d, J = 2.34 Hz, 2H), 2.45 (t, J = 2.34 Hz, 1H). ^{13}C NMR (100 MHz, $CDCl_3$) δ (ppm): 159.50, 149.47, 136.82, 122.81, 121.97, 83.92, 79.30, 75.20, 56.56.

3: *2,2′-((4-iodobenzyloxy)methylene)dipyridine*

^{1}H NMR (400 MHz, $CDCl_3$) δ (ppm): 8.56 (d, J = 6.15 Hz, 2H), 7.71 (t, J = 7.91 Hz, 2H), 7.65 (d, J = 8.79 Hz, 2H), 7.60 (d, J = 7.91 Hz, 2H), 7.19 (t, J = 6.40 Hz, 2H), 7.11 (d, J = 7.91 Hz, 2H), 5.74 (s, 1H), 4.58 (s, 2H). ^{13}C NMR (100 MHz, $CDCl_3$) δ (ppm): 160.15, 149.48, 137.62, 136.73, 129.78, 122.72, 121.64, 93.45, 85.45, 70.78.
